# Overexpression of Wild-Type but Not C134W Mutant FOXL2 Enhances GnRH-Induced Cell Apoptosis by Increasing GnRH Receptor Expression in Human Granulosa Cell Tumors

**DOI:** 10.1371/journal.pone.0055099

**Published:** 2013-01-23

**Authors:** Jung-Chien Cheng, Christian Klausen, Peter C. K. Leung

**Affiliations:** Department of Obstetrics and Gynaecology, Child and Family Research Institute, University of British Columbia, Vancouver, British Columbia, Canada; Institut de Génomique Fonctionnelle de Lyon, France

## Abstract

The etiology of granulosa cell tumors (GCTs) is largely unknown. The primary mode of treatment is surgical, however not all women are cured by surgery alone. Thus, it is important to develop improved treatments through a greater understanding of the molecular mechanisms that contribute to this disease. Recently, it has been shown that a *FOXL2* 402C>G (C134W) mutation is present in 97% of human adult-type GCTs, suggesting an important role for this mutation in the development of GCTs. We have shown previously that gonadotropin-releasing hormone (GnRH)-I and -II induce apoptosis in cultured normal human granulosa cells. Moreover, it has been reported that FOXL2 can bind to the promoter of the mouse GnRH receptor gene and regulate its transcription. Thus, we hypothesized that C134W mutant FOXL2 could modulate the pro-apoptotic effects of GnRH via aberrant regulation of GnRH receptor levels. Using KGN cells, a human GCT-derived cell line which harbors the *FOXL2* 402C>G mutation, we show that treatment with GnRH-I and -II induces cell apoptosis, and that small interfering RNA-mediated depletion of GnRH receptor abolishes these effects. Overexpression of wild-type FOXL2 increases both mRNA and protein levels of GnRH receptor and consequently enhances GnRH-induced apoptosis. Importantly, neither the expression levels of GnRH receptor nor GnRH-induced apoptosis were affected by overexpression of the C134W mutant FOXL2. Interestingly, knockdown of endogenous FOXL2 down-regulates GnRHR expression in normal human granulosa cells with wild-type FOXL2, but not in KGN cells. These results suggest that the *FOXL2* 402C>G mutation may contribute to the development of human adult-type GCTs by reducing the expression of GnRH receptor, thus conferring resistance to GnRH-induced cell apoptosis.

## Introduction

Ovarian cancer is the most lethal gynecological malignancy in the Western world. Granulosa cell tumors (GCTs) account for approximately 5% of all ovarian cancers and occur at a frequency of 1 in 100,000. The peak incidence for GCTs is between 50 and 60 years of age [1]. To date, GCTs are a poorly understood cancer whose pathogenesis is unknown and for which there is no effective treatment beyond primary surgery.

Gonadotropin-releasing hormone (GnRH) is a hypothalamic neuropeptide that functions as a key neuroendocrine regulator of the hypothalamic-pituitary-gonadal axis. Humans possess two distinct forms of GnRH, termed GnRH-I and GnRH-II, as well as one conventional GnRH receptor subtype (type I GnRHR). It is generally accepted that both forms of GnRH exert their biological effects by binding to the type I GnRHR [2]. We have previously demonstrated that GnRHR and both forms of GnRH are expressed in human granulosa-luteal cells [3], [4]. These data suggest putative regulatory roles for GnRHs in the development and function of ovarian follicles and/or corpus luteum. Indeed, we have shown that GnRH-I and -II can induce apoptosis in human granulosa cells [5]. However, whether GnRH can trigger apoptosis in human GCTs is still unknown.

The *FOXL2* gene encodes a member of the forkhead/winged-helix family of transcription factors which contains a highly conserved forkhead DNA binding domain [6]. Germline loss-of-function mutations in *FOXL2* are associated with BPES (blepharophimosis ptosis epicanthus inversus syndrome) which is characterized by shortening of the horizontal orbital fissure (blepharophimosis), congenital ptosis and epicanthus inversus [6], [7]. BPES is categorized into two types: Type I with premature ovarian failure and Type II with normal fertility [8]. Truncating mutations of *FOXL2* generally lead to Type I disease whereas frameshifts or duplications downstream of the forkhead domain lead to Type II disease [9]. Recent studies have identified a *FOXL2* 402C>G (C134W) mutation that is present in 97% of human adult-type GCTs, but absent in unrelated ovarian or breast tumors [10]. These findings strongly suggest an important role for FOXL2 in granulosa cell tumorigenesis. Indeed, overexpression of FOXL2 in rat granulosa cells results in reduced cell viability [11], and C134W mutant FOXL2 is a weaker inducer of apoptosis in human GCT-derived KGN cells [12].

FOXL2 can act as either a transcriptional activator or repressor to regulate variety of genes [13]. Interestingly, it has been shown that the mouse *Gnrhr* gene can be activated by FOXL2 in mouse pituitary cells [14]. Despite sharing a high degree of homology with the mouse sequence, we have shown that the human GnRHR promoter differs from the mouse promoter with respect to involvement of various transcriptional motifs, and that its regulation varies among human tissues [4]. Thus, it is not known whether FOXL2 is involved in the transcriptional regulation of *GnRHR* in human granulosa cells.

Human GCT-derived KGN cells are heterozygous for the somatic *FOXL2* 402C>G mutation and are a useful model to study the biology of GCTs [15]. In the present study, we report for the first time that GnRHR is expressed in KGN cells and that treatment with GnRH-I and GnRH-II induces apoptosis in KGN cells. Overexpression wild-type FOXL2, but not C134W mutant FOXL2, increases GnRHR expression and enhances GnRH-induced cell apoptosis. These results suggest that the 402C>G *FOXL2* gene mutation may contribute to human granulosa cell tumorigenesis by diminishing GnRH/GnRHR-induced cell apoptosis.

## Materials and Methods

### Cell culture

The previously established human GCT-derived KGN cells [16] were obtained from the RIKEN Bioresource Center Cell Bank with the permission of Dr. Toshihiko Yanase http://www.rbej.com/content/8/1/61/ – ins4 (Department of Medicine and Bioregulatory Science, Kyushu University, Japan). This cell line was derived from a patient with an invasive ovarian granulosa cell tumor and it maintains many of the physiological characteristics of normal granulosa cells, including the expression of functional FSH receptor and the expression of aromatase [16]. SV40 large T antigen immortalized human granulosa-luteal (hGL) cells were established previously by our group [17]. KGN cells and immortalized hGL cells were grown in DMEM/F12 medium (Sigma-Aldrich, Oakville, ON) supplemented with 10% fetal bovine serum (FBS; Hyclone Laboratories Inc., Logan, UT). Cultures were maintained at 37°C in a humidified atmosphere of 5% CO_2_ in air.

### Antibodies and reagents

The mouse monoclonal anti-GnRHR antibody was obtained from NeoMarkers (Fremont, CA). Goat polyclonal anti-FOXL2 antibody was obtained from IMGENEX (San Diego, CA). Rabbit polyclonal anti-caspase-3 antibody was obtained from Cell Signaling (Danvers, MA). The goat polyclonal anti-actin antibody was obtained from Santa Cruz Biotechnology (Santa Cruz, CA). Horseradish peroxidase-conjugated goat anti-rabbit and donkey anti-goat IgGs were obtained from Bio-Rad Laboratories (Hercules, CA) and Santa Cruz Biotechnology, respectively. GnRH-I agonist was obtained from Sigma and GnRH-II agonist was obtained from Bachem (Torrance, CA).

### Plasmid constructs

The pcDNA3 expression vector encoding full-length wild-type human FOXL2 was kindly provided by Dr. Aaron Hsueh http://www.rbej.com/content/8/1/61/ – ins4(Department of Obstetrics and Gynecology, Stanford University) [18]. The 402C>G (C134W) mutation was generated using the Quickchange II XL site directed mutagenesis kit (Stratagene, La Jolla, CA) with the wild-type FOXL2 construct as template and the following primers: 5′ -CTG GAC GCT GGA CCC GGC CTG CGA AGA CAT GTT CGA GAA GGG C-3′ (sense) and 5′ -GCC CTT CTC GAA CAT GTC TTC CCA GGC CGG GTC CAG CGT CCA G-3′ (antisense). Mutated constructs were sequenced to verify that only the desired mutation was present. To overexpress wild-type or C134W mutant FOXL2, cells were transfected with 1 µg of plasmid DNA using Lipofectamine 2000 (Invitrogen, Burlington, ON) and empty pcDNA3 plasmid was used as the transfection control.

### Small interfering RNA transfection

To knockdown endogenous GnRHR and FOXL2, cells were transfected with 50 nM ON-TARGETplus SMARTpool GnRHR or FOXL2 siRNA (Dharmacon, Lafayette, CO) using Lipofectamine RNAiMAX (Invitrogen). Wild-type and mutant FOXL2 mRNAs were equally targeted by all FOXL2 siRNA duplexes. ON-TARGETplus Non-Targeting Pool siRNA (Dharmacon) was used as a transfection control and knockdown efficiency was examined by reverse transcription quantitative real-time PCR (RT-qPCR) or Western blot analysis.

### MTT assay

MTT (3-(4,5-Dimethylthiazol-2-yl)-2,5-diphenyltetrazolium bromide; Sigma) assay was used to determine cell viability. KGN cells were seeded in 24-well plates (1×10^4^ cells/well in 500 µL of medium) and on the following day were treated every 24 hr with vehicle, GnRH-I or GnRH-II in medium containing 1% of FBS. MTT was added to a final concentration of 0.5 mg/mL, the cells were incubated for 4 hr and the medium was removed. DMSO was added to each well and absorbances were measured at 490 nm using a microplate reader.

### Western blots

Equal amounts of protein were separated by SDS-PAGE and transferred to PVDF membranes. Following blocking with Tris-buffered saline containing 5% non-fat dry milk for 1 hr, membranes were incubated overnight at 4°C with primary antibodies followed by incubation with secondary antibody. Immunoreactive bands were detected with enhanced chemiluminescent substrate. Membranes were stripped with stripping buffer (62.5 mM Tris, 10 mM DTT, 2% SDS, pH 6.7) at 50°C for 30 min, and reprobed with anti-actin as a loading control.

### Reverse transcription quantitative real-time PCR (RT-qPCR)

Total RNA was extracted using TRIzol reagent (Invitrogen) according to the manufacturer's instructions. Reverse transcription was performed with 3 µg RNA, random primers and M-MLV reverse transcriptase (Promega, Madison, WI). Each 20 μL RT-qPCR reaction contained 1X SYBR Green PCR Master Mix (Applied Biosystems), 120 ng cDNA and 500 nM of each specific primer. The primers used were as follows: GnRHR, 5′- ACC GCT CCC TGG CTA TCA C-3′ (sense) and 5′- GAC TGT CCG ACT TTG CTG TTG CT -3′ (antisense); FOXL2, 5′- CAT GTT CGA GAA GGG CAA CT -3′ (sense) and 5′-AGG AAG CCA GAC TGC AGG TA-3′ (antisense) and GAPDH, 5′-GAG TCA ACG GAT TTG GTC GT-3′ (sense) and 5′-GAC AAG CTT CCC GTT CTC AG-3′ (antisense). RT-qPCR was performed on an Applied Biosystems 7300 Real-Time PCR System (Perkin-Elmer) equipped with a 96-well optical reaction plate. All RT-qPCR experiments were run in triplicate and a mean value was used for the determination of mRNA levels. Relative quantification of mRNA levels was performed using the comparative Cq method with GAPDH as the reference gene and with the formula 2^–ΔΔCq^.

### Statistical analysis

Results are presented as the mean ± SEM of at least three independent experiments. For experiments involving only two groups, the data were analyzed by Excel with a Two-Sample *t*-test assuming unequal variances. Multiple group comparisons were analyzed by one-way ANOVA followed by Tukey's multiple comparison test using PRISM software. Significant differences were defined as *p*<0.05.

## Results

### GnRH-I and GnRH-II decrease cell viability via GnRHR

**Figure 1:**
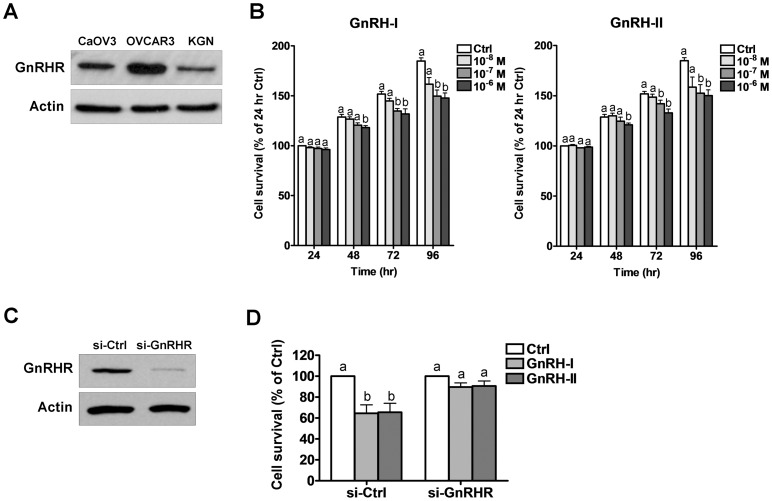
GnRH-I and GnRH-II reduce cell viability via GnRH receptor (GnRHR). A, Total GnRHR protein levels were analyzed by Western blot. Two ovarian cancer cell lines, CaOV3 and OVCAR3, were used as positive controls. B, KGN cells were treated every 24 hr with GnRH-I or GnRH-II (10^−6^–10^−8^ M) in medium containing 1% FBS for 24–96 hr, and MTT assay was used to measure cell viability. The results were analyzed at each time point. C, KGN cells were transfected for 72 hr with 50 nM control siRNA (si-Ctrl) or GnRHR siRNA (si-GnRHR) and GnRHR protein levels were examined by Western blot. D, KGN cells were transfected with control siRNA (si-Ctrl) or GnRHR siRNA (si-GnRHR) for 24 hr and treated with GnRH-I or GnRH-II (10^−7^ M) for a further 96 hr, and cell viability was examined by MTT assay. Results are expressed as mean ± SEM of at least three independent experiments. Values without a common letter are significantly different (p<0.05).

To investigate the effects of GnRH on cell apoptosis we first confirmed the expression of GnRHR in KGN cells. Two human ovarian cancer cell lines, CaOV3 and OVCAR3, were used as positive controls because we have previously demonstrated that they express GnRHR [19]. Total cell lysates were collected and GnRHR protein levels were examined by Western blot. As shown in Figure 1A, GnRHR expression in KGN cells is similar to that of two ovarian cancer cell lines with demonstrated responsiveness to GnRH [19].

We have shown that both forms of GnRH can induce cell growth inhibition in human ovarian and endometrial cancer cells [20], [21]. To examine the anti-proliferative effects of GnRH on KGN cells, we treated the cells with different concentrations of GnRH-I or GnRH-II for 24, 48, 72 or 96 hr, and cell viability was examined by MTT assay. As shown in Figure 1B, cell viability was significantly reduced by treatment with GnRH-I or GnRH-II (10^−6^ and 10^−7^ M) for 72 and 96 hr, and both forms of GnRH have similar effects. To determine whether the effects of GnRH on cell viability are mediated by GnRHR, specific siRNA was used to knockdown endogenous GnRHR in KGN cells prior to treatment with GnRH. Transfection with GnRHR siRNA significantly reduced GnRHR protein levels (Figure 1C) and abolished the effects of GnRH-I or GnRH-II (10^−7^ M, 96 hr) on cell viability (Figure 1D).

### GnRH-I and GnRH-II induce cell apoptosis

**Figure 2:**
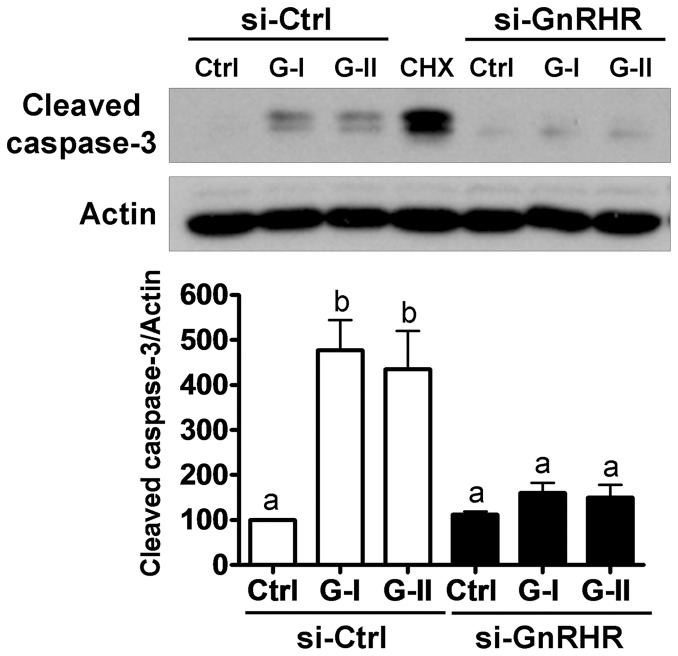
GnRH-I and GnRH-II induce cell apoptosis via GnRHR. KGN cells were transfected with 50 nM control siRNA (si-Ctrl) or GnRHR siRNA (si-GnRHR) for 24 hr and then treated with GnRH-I (G-I) or GnRH-II (G-II) (10^−7^ M) for a further 72 hr. Cycloheximide (CHX; 100 µM for 24 hr) was used as a positive control and cleaved caspase-3 protein levels were examined by Western blot. Results are expressed as mean ± SEM of at least three independent experiments. Values without a common letter are significantly different (p<0.05).

GnRH-induced reductions in the number of viable cells may result from the direct inhibition of cell proliferation or the induction of cell death. Having previously demonstrated GnRH-induced apoptosis in human immortalized granulosa cells [5], we therefore examined the effect of GnRH on KGN cell apoptosis by measuring the levels of cleaved caspase-3 by Western blot. As shown in Figure 2, treatment with GnRH-I or GnRH-II increased cleaved caspase-3 protein levels, as did the positive control, cycloheximide. Similar to the results from the MTT assay, GnRH-I and GnRH-II-induced increases in cleaved caspase-3 were abolished by siRNA-mediated knockdown of GnRHR. These results indicate that GnRH induces apoptosis in human granulosa tumor cells via type I GnRHR.

### Overexpression of wild-type but not C134W mutant FOXL2 increases GnRHR expression

**Figure 3:**
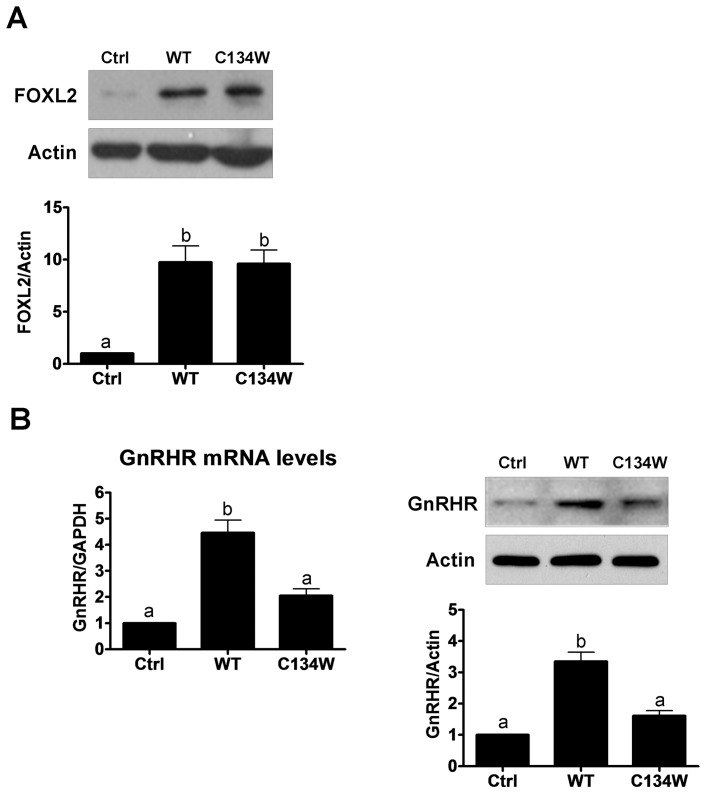
Overexpression wild-type but not C134W mutant FOXL2 increases GnRH receptor (GnRHR) expression. KGN cells were transfected with empty pcDNA3 plasmid (Ctrl), wild-type FOXL2 (WT) or C134W mutant FOXL2 (C134W). A, Western blot was used to measure FOXL2 protein (48 hr) levels in transfected cells. B, RT-qPCR and Western blot were used to measure GnRHR mRNA (24 hr) and protein (48 hr) levels in transfected cells. Results are expressed as mean ± SEM of at least three independent experiments. Values without a common letter are significantly different (p<0.05).

Although it has been reported that FOXL2 can increase GnRHR expression in mouse pituitary cells [14], whether or not the same is true for human tissues is unknown. Thus, KGN cells were transiently transfected with empty vector, wild-type FOXL2 or C134W mutant FOXL2 to investigate the effects of FOXL2 on GnRHR expression. Overexpression of wild-type and mutant FOXL2 was confirmed by Western blot analysis after 48 hr transfection (Figure 3A). Transfection with wild-type FOXL2 significantly increased GnRHR mRNA and protein levels compared to empty vector control. In contrast, overexpression of C134W mutant FOXL2 did not induce significant changes the mRNA and protein levels of GnRHR (Figure 3B). These results suggest that wild-type FOXL2 can regulate GnRHR expression in KGN cells, but that C134W mutant FOXL2 does not.

### GnRH-induced apoptosis is enhanced by wild-type but not C134W mutant FOXL2

**Figure 4:**
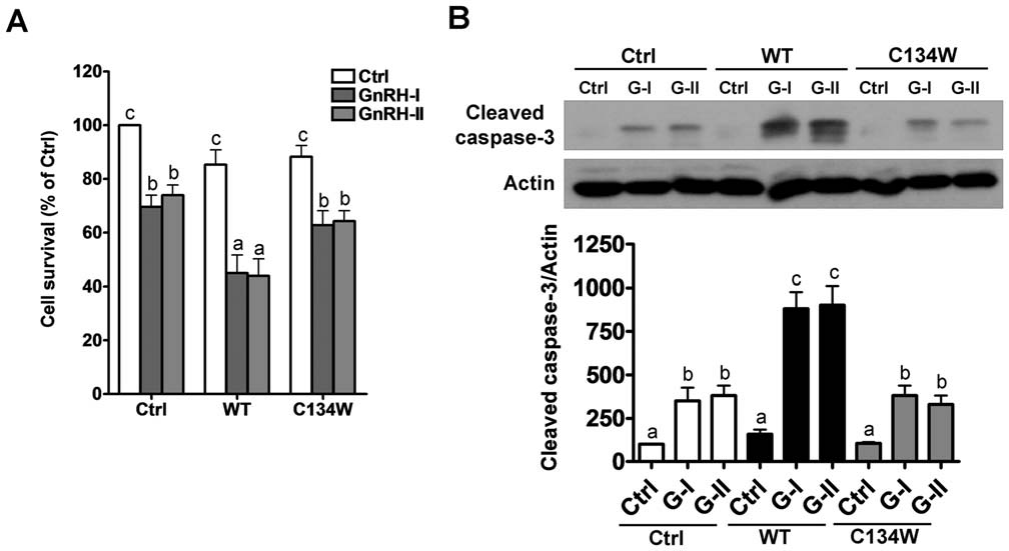
GnRH-induced apoptosis is enhanced by wild-type but not C134W mutant FOXL2. A, KGN cells were transfected with empty pcDNA3 plasmid (Ctrl), wild-type FOXL2 (WT) or C134W mutant FOXL2 (C134W) for 24 hr and then treated with GnRH-I or GnRH-II (10^−7^ M) for 96 hr. Cell viability was examined by MTT assay. B, KGN cells were transfected as described in (A) and then treated with GnRH-I (G-I) or GnRH-II (G-II) (10^−7^ M) for 72 hr. Cleaved caspase-3 protein levels were measured by Western blot. Results are expressed as mean ± SEM of at least three independent experiments. Values without a common letter are significantly different (p<0.05).

To examine whether FOXL2-mediated changes in GnRHR expression affect GnRH-induced cell apoptosis, KGN cells were transfected with empty vector, wild-type FOXL2 or C134W mutant FOXL2 and then treated with vehicle, GnRH-I or GnRH-II. As shown in Figure 4A, GnRH-induced reductions in cell viability were enhanced in cells transfected with wild-type FOXL2 compared to empty vector control. In contrast, transfection with C134W mutant FOXL2 did not alter the effects of GnRH on cell viability, as assessed by MTT assay (Figure 4A). In agreement with these findings, Western blot analysis of cleaved caspase-3 levels confirmed the enhancement of GnRH-induced apoptosis in cells transfected with wild-type but not C134W mutant FOXL2 (Figure 4B).

### Knockdown of FOXL2 in KGN cells does not alter GnRHR expression levels

**Figure 5:**
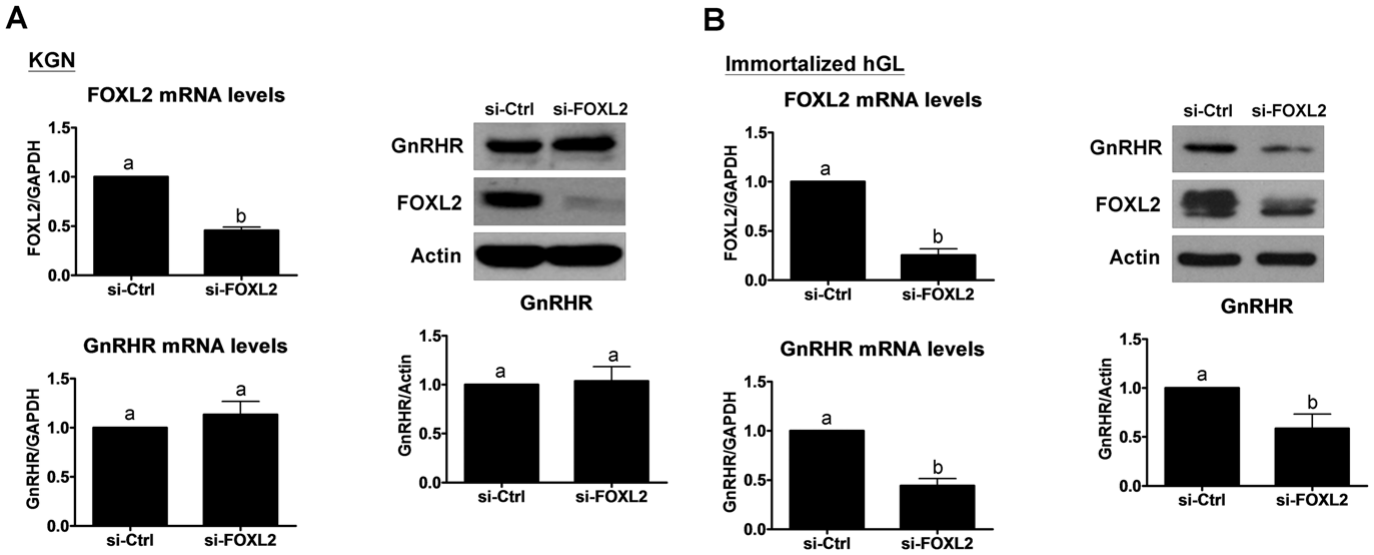
Knockdown of endogenous FOXL2 down-regulates GnRH receptor (GnRHR) in immortalized human granulosa-luteal cells but not KGN cells. A, KGN cells were transfected with 50 nM control siRNA (si-Ctrl) or FOXL2 siRNA (si-FOXL2). RT-qPCR and Western blot were used to measure the mRNA (left panel) and protein (right panel) levels of FOXL2 and GnRHR. B, Immortalized human granulosa-luetal cells (hGL) were transfected with 50 nM control siRNA (si-Ctrl) or FOXL2 siRNA (si-FOXL2). RT-qPCR and Western blot were used to measure the mRNA (left panel) and protein (right panel) levels of FOXL2 and GnRHR. Results are expressed as the mean ± SEM of at least three independent experiments. Values without a common letter are significantly different (p<0.05).

Having established that forced-expression of wild-type FOXL2 can regulate GnRHR expression; we next investigated the effects of FOXL2 knockdown on GnRHR since KGN cells are heterozygous for the C134W mutation. As shown in Figure 5A, treatment with FOXL2 siRNA significantly down-regulated FOXL2 mRNA levels whereas GnRHR mRNA levels remained unchanged. Likewise, treatment with FOXL2 siRNA did not alter GnRHR protein levels in spite of profound reductions in FOXL2 protein levels. To further examine the role of endogenous FOXL2 in the regulation of GnRHR, we investigated the effects of FOXL2 knockdown on GnRHR mRNA and protein levels in immortalized human granulosa-luteal (hGL) cells with wild-type FOXL2. In contrast to the results obtained in KGN cells, FOXL2 siRNA treatment significantly reduced basal GnRHR mRNA and protein levels in immortalized hGL cells (Figure 5B). These results show that endogenous FOXL2 maintains elevated GnRHR levels in normal human granulosa cells, but not in heterozygous mutant KGN cells.

## Discussion

Due to the low incidence of GCTs, representing 2–5% of all ovarian cancers, little is known about their pathogenesis. GCTs are generally thought to have a better prognosis than epithelial tumors, however recurrent disease still claims the lives of 20–30% of patients diagnosed with advanced tumors [1]. Recent studies identifying a somatic *FOXL2* 402C>G (C134W) mutation present in virtually all morphologically identified adult-type GCTs strongly suggest the involvement of FOXL2 in the pathogenesis of adult-type GCTs [10]. Yet, little is known about the function of FOXL2 in human granulosa cells or the biological implications of the 402C>G mutation. In a *Foxl2^lacZ^* homozygous mutant mouse model, the transition from a squamous to cuboidal form of granulosa cell is blocked leading to the absence of secondary follicles, suggesting that Foxl2 is required for granulosa cell differentiation and maintenance of ovarian function [22].

Pro-apoptotic effects of GnRH-I and GnRH-II have been reported in cell lines derived from several human malignancies [2]. In rats, treatment with GnRH-I induces apoptosis in granulosa cells isolated from preovulatory follicles [23]. We have shown that treatment with GnRH-I or GnRH-II stimulates human immortalized granulosa cells to undergo apoptosis [5]. However, whether human GCTs express GnRHR or display pro-apoptotic responses to GnRH remains unknown. We now demonstrate that GnRH-I and GnRH-II induce cell apoptosis in human GCT-derived KGN cells. In addition, we used GnRHR siRNA to show that the apoptotic effects of GnRH are mediated by type I GnRHR. These results indicate that GnRH induces cell apoptosis in both normal and malignant granulosa cells.

It has been shown that AP-1, Smad3, Smad4 and Foxl2 can bind to the Gnrhr activating sequence (GRAS) and activate transcription of a Gnrhr promoter-luciferase construct in mouse αT3-1 gonadotrope cells [14]. Likewise, overexpression of Foxl2 resulted in a significant increase in murine Gnrhr promoter activity in mouse KK1 granulosa cells [24]. However, despite sharing a high degree of homology with the mouse sequence, the human gene possesses neither the AP-1 nor the GRAS site in the corresponding positions along the proximal promoter of *GnRHR* [25]. Studies suggest that transcription factors such as Oct-1, CREB, GATA-2, GATA-3, and c-Jun/c-Fos contribute to the transcriptional regulation of human *GnRHR*, however, the role of FOXL2 has not been investigated [2]. Here, for the first time, we show that overexpression of wild-type, but not C134W mutant, FOXL2 increases GnRHR mRNA and protein levels in human GCT cells. Our FOXL2 knockdown experiments show that basal GnRHR levels are maintained by wild-type FOXL2 in normal human granulosa cells. In contrast, depletion of FOXL2 in heterozygous mutant KGN cells did not affect GnRHR levels. Thus, our results reveal important differences in the regulation of basal GnRHR levels by FOXL2 between wild-type and mutant cells.

In addition to demonstrating that wild-type FOXL2 regulates human GnRHR expression, we also show that C134W mutant FOXL2 is deficient in this capacity. Since we have demonstrated the pro-apoptotic effects of GnRH in human GCT cells, this might suggest a scenario where the mutation of *FOXL2* leads to reduced GnRHR expression and consequent resistance to GnRH-induced apoptosis. Recently, Kim *et al*. demonstrated that overexpression of wild-type FOXL2 induced apoptosis and increased cleaved caspase-3 levels in KGN cells [12]. Moreover, they also found that C134W mutant FOXL2 was a weaker inducer of apoptosis, likely because of its reduced ability to induce the expression of tumor necrosis factor receptor 1 (TNFR1) and FAS. Thus by up-regulating key pro-apoptotic receptors (GnRHR, TNFR1 and FAS), wild-type FOXL2 contributes to the homeostatic balance of human granulosa cells, and its mutation may result in resistance to apoptosis and lead to granulosa cell tumorigenesis.

In our study, overexpression of wild-type FOXL2 alone did not significantly affect cell viability or cleaved caspase-3 levels. However, it is important to consider that the time-points examined in our study (96 hr) differed significantly from those of Kim *et al*. (24 hr) [12]. Indeed, the purpose of our study was to investigate the effects of FOXL2 on GnRH-induced cell apoptosis (requiring prolonged treatment with GnRH), rather than the pro-apoptotic effects of FOXL2 itself. Importantly, accumulating evidence indicates that wild-type and mutant FOXL2 differentially regulate a variety of genes involved in cell proliferation and apoptosis. Thus, in addition to effects on pro-apoptotic ligands and receptors, mutant FOXL2 may contribute to granulosa cell tumorigenesis by de-regulating steroidogenesis [26], TGF-β signaling [27] or interactions with various partner proteins [28].

Exactly how the substitution of tryptophan for cysteine at position 134 alters the function of FOXL2 remains unclear. The DNA-binding specificity of forkhead proteins is partially determined by highly variable wing regions (W1 and W2) whose conformations contribute to the precise positioning of the recognition helix [29]. The C134W mutation is predicted to affect the W2 wing region which suggests that it may alter the recognition of specific promoters. However, *in silico* homology modeling based on the crystal structures of FOXK2 or FOXP2 suggests that the substitution may disrupt protein-protein interactions rather than DNA binding [10], [30]. Recent studies also indicate that the C134W mutation does not affect the localization, mobility and transactivation abilities of FOXL2 [30]. Thus, it has been proposed that the C134W mutation is neither a dominant-negative nor a loss-of-function mutation [30]. Yet, virtually all studies examining the impact of the C134W mutation have measured promoter-luciferase activities rather than endogenous gene expression. For instance, 3xGRAS-luciferase activity was higher in KGN cells transfected with C134W mutant FOXL2 than wild-type FOXL2, whereas the opposite was observed in HeLa cells [30]. Our studies are the first to examine the effects of FOXL2 on endogenous GnRHR expression, and they show that C134W mutant FOXL2 is not an effective inducer of GnRHR expression in KGN cells. Moreover, our FOXL2 siRNA results are consistent with putative dominant-negative effects of C134W mutant FOXL2, and suggest that the expression profile of heterozygous KGN cells is biased towards the mutant allele, at least as it relates to GnRHR expression. Interestingly, it has been shown that a Smad3-Foxl2 heterodimer can bind to the GRAS sequence of the murine Gnrhr promoter [14] and subsequently, it was proposed that the C134W mutation may alter interactions between Foxl2 and Smad3 [30]. However, at present it is not known if Smad3 participates in FOXL2-induced human GnRHR expression, or whether the C134W mutation alters interactions between these two proteins. It is likely that FOXL2 interacts with many transcriptional regulators to orchestrate tissue specific gene expression. Thus, future studies investigating the roles of wild-type and mutant FOXL2 in the regulation of endogenous granulosa cell gene expression should provide important insights into the pathogenesis of adult-type GCTs.

In summary, our studies demonstrate that GnRH exerts pro-apoptotic effects on human GCT cells, and suggest that autocrine GnRH/GnRHR signaling may contribute to normal human granulosa cell function. Moreover, we show that overexpression of wild-type FOXL2 up-regulates GnRHR and enhances GnRH-induced apoptosis, whereas C134W mutant FOXL2 does not. Thus, the *FOXL2* 402C>G mutation may lead to the development of human adult-type GCTs by reducing the expression of GnRHR, thus conferring resistance to GnRH-induced apoptosis and enhancing proliferative capacity.
